# Comparison of Empirical Mode Decomposition and Singular Spectrum Analysis for Quick and Robust Detection of Aerodynamic Instabilities in Centrifugal Compressors †

**DOI:** 10.3390/s22052063

**Published:** 2022-03-07

**Authors:** Mateusz Stajuda, David García Cava, Grzegorz Liśkiewicz

**Affiliations:** 1 School of Engineering, Institute for Infrastructure and Environment, University of Edinburgh, Thomas Bayes Road, Edinburgh EH9 3FG, UK; david.garcia@ed.ac.uk; 2 Institute of Turbomachinery, Lodz University of Technology, Wólczanska 219/221, 90-924 Lodz, Poland; grzegorz.liskiewicz@p.lodz.pl

**Keywords:** signal processing, centrifugal compressor, surge, inlet recirculation, EMD, SSA

## Abstract

Aerodynamic instabilities in centrifugal compressors are dangerous phenomena affecting machine efficiency and in severe cases leading to failure of the compressing system. Quick and robust instability detection during compressor operation is a challenge of utmost importance from an economical and safety point of view. Rapid indication of instabilities can be obtained using a pressure signal from the compressor. Detection of aerodynamic instabilities using pressure signal results in specific challenges, as the signal is often highly contaminated with noise, which can influence the performance of detection methods. The aim of this study is to investigate and compare the performance of two non-linear signal processing methods—Empirical Mode Decomposition (EMD) and Singular Spectrum Analysis (SSA)—for aerodynamic instability detection. Two instabilities of different character, local—inlet recirculation and global—surge, are considered. The comparison focuses on the robustness, sensitivity and pace of detection—crucial parameters for a successful detection method. It is shown that both EMD and SSA perform similarly for the analysed machine, despite different underlying principles of the methods. Both EMD and SSA have great potential for instabilities detection, but tuning of their parameters is important for robust detection.

## 1. Introduction

Centrifugal compressors are machines of great importance for a wide range industries, operating in petrol engines, turboshaft engines and industrial plants of various kinds [1]. The compressor operating range is limited by choke for high mass flow rates and the appearance of aerodynamic instabilities at low mass flow rates [2]. The compressor peak efficiency region is adjacent to the unstable region, therefore, it is not uncommon for instabilities to appear during standard machine operation, when a slight change in external conditions takes place. There exist a number of well-described instabilities, such as inlet recirculation, rotating stall or surge [3,4]. Inlet recirculation and rotating stall, being local instabilities, are often predecessors of a global instability—surge [5]. The instabilities in centrifugal compressor may vary in effect, ranging from drop in efficiency for inlet recirculation [6], through non-synchronous vibrations introduced by rotating stall [7], up to an abrupt destruction of a compressor in case of surge [3].

The field of instabilities detection is still in development, focusing more and more on application of data-driven techniques. The most commonly presented methods were based on Wavelet Transform [8,9] which performed well for selected instabilities. However, wavelet transform requires a choice of mother wavelet, which is not always trivial. One of the major drawbacks of WT is that it uses a fixed decomposition scale for analysis and does not take the signal’s characteristics into consideration [10]. It also suffers from a leakage problem [11] that arises due to limited length of the wavelet function. Rotating stall can be detected by taking advantage of bifurcation theory [12], but the method has not been extended to detection of other instabilities. Attempts to use singular value decomposition (SVD) in compressor stability detection were also made [13], displaying valuable results for surge and stall.

The signature of instabilities in the pressure signal is different for each phenomenon. With inlet recirculation, a broadband noise without a dominating frequency is observed. For rotating stall, a periodic component is present in the signal, but the period may differ depending on the conditions as the number of stall cells may differ in time. For deep surge, a periodic excitation is present. Therefore, the detection method that can be useful for all of those unstable phenomena has to be universal.

A number of different types of non-linear signal analysis exist, some of which could prove advantageous for detection of instabilities, such as superlets [14], intrinsic timescale decomposition [15], variational mode decomposition [16] or other recent techniques developed in the field of radio communication [17,18] and radars, where the information of interest has to be extracted from noise or clutter [19]. Alternatively, a number of machine learning techniques have been created, which can be used for the purpose of instabilities detection, as they are used for fault diagnosis [20]. However, use of machine learning requires a large number of data points and often lacks interpretability, which can be offered by classical feature extraction techniques.

A promising solution for comprehensive aerodynamic instabilities detection is offered by Empirical Mode Decomposition (EMD) and Singular Spectrum Analysis (SSA). Both of those methods are established decomposition techniques performing well in numerous applications. EMD was used for bearings fault detection [21], machining process monitoring [22,23] or financial predictions [24]. SSA was demonstrated to provide valuable insights in the same fields [25,26,27]. Recently, it was shown that SSA can be used for detection of instabilities such as surge [28] and inlet recirculation [29] which can be regarded as a surge predecessor [30]. The same capabilities were demonstrated for EMD [31]. Neither of those studies considered the influence of length of the input signal or stochastic character of the pressure, as they both used long portions to demonstrate the potential of the methods. The choice of EMD and SSA as the methods for comparison resulted from their proven performance for compressor instability detection. A preliminary study on the pace of detection for EMD and SSA was conducted by the authors [32], which showed that the method could provide quick and robust indication of the condition. This article builds upon previous study to provide quantification of methods performance, considering the stochastic nature of the signal, give more insight into the method operation for compressor pressure signal and deliver more thoughtful conclusions that can be applied when building an instabilities detection system.

The requirement of application to the centrifugal compressor instability detection system is that the methods must be responsive or work in real time to ensure safety of operation. Thus, a step towards implementation of SSA and EMD into an instabilities detection system is to validate their potential for robust detection of instabilities based on a short signal portions. The pace of detection depends on two aspects. The first aspect, referred to hereafter as sensitivity, is the signal length needed for robust detection, expressed in number of data points. The shorter a signal portion allowing for a robust detection, the quicker a method can react to a change in conditions as less acquisition time is needed. Sensitivity might differ between methods as they might rely on different signal features. The other aspect is data processing time needed for obtaining an indication of conditions. Different methods may have a different computational cost, resulting in a longer or shorter time required for processing of the data. If the computation time is similar or shorter than the acquisition time, the detection system can operate continuously in real time as before the subsequent signal portion is acquired, the previous one was already analysed and the conditions were defined. Processing time is highly dependent on the method algorithm, but also on its implementation, consisting of both software and hardware. Implementation aspects were not optimized in this work and the methods were benchmarked using a predefined setup.

The main motivation of this study is evaluation of EMD and SSA for application in a real-time centrifugal compressor instability detection system. It was shown in previous studies [29,31] that both of the methods can be used to extract the features of instabilities from a pressure signal of a centrifugal compressor, but neither of those studies focused on the pace of detection. Through analysis of the methods’ sensitivity, understanding the behaviour of components for varying parameters, it is possible to evaluate the potential of EMD and SSA for real-time instability detection. The parametric study can also demonstrate which parameters should be optimized and which have little influence on the detection performance. Based on the analysis of the components, the advantages and limitations of the methods are presented.

## 2. Materials and Methods

### 2.1. Decomposition Methods

Decomposition methods can be used on the input signal to filter the unwanted components and extract others that can highlight changes in the system of interest. The performance of the methods differs and might be specific to the area of application due to different underlying physics of the changes to be detected and character of the input signal. Understanding the rationale of method operation and why it performs well for a particular case is essential for generalization of the findings to a whole class of problems. In the next sections, the basics of EMD and SSA are introduced.

#### 2.1.1. Empirical Mode Decomposition

EMD is based on the assumption that signal consists of a sum of simple oscillatory modes—intrinsic mode functions (IMFs)—and a residue [33] that can be extracted with EMD. The method was first introduced in a work by Huang [34], in which a detailed mathematical explanation can be found. EMD is able to deal with non-stationary and non-linear due to its direct and adaptive algorithm, with a decomposition base derived from the data. IMFs, as a result of their derivation, can reflect changes in both amplitude and frequency of phenomena in the analyzed signal. The IMFs are extracted with an empirical procedure in an iterative manner, making use of the envelope of the signal. To do so, all the local extrema are connected by cubic spline, creating the envelope. The first component of decomposition $h_{1}$ is obtained from Equation (1), where x(t) is original time series, $m_{1}$ is the envelope mean.

(1) $$h_{1}\left(t\right)=x\left(t\right)-m_{1}\left(t\right)$$

Then, it is checked if a stoppage criterion is satisfied by $h_{1}\left(t\right)$. There exist a number of different criteria, with different influence on the produced IMFs [35]. A Cauchy type criterion is used, as it was originally introduced by Huang [34] and is the most basic among all of the criteria. This criterion is based on difference between two subsequent sifting iterations and can be presented as in Equation (2). This criterion in several code implementations can be overridden by putting explicitly the number of sifting iterations that are to be made.

(2) $$SD_{n}=\frac{\sum_{t=1}^{N}{\left|h_{n-1}\left(t\right)-h_{n}\left(t\right)\right|}^{2}}{\sum_{t=1}^{N}h_{n-1}^{2}\left(t\right)}$$

If the stoppage criterion is not met and maximum number of iterations is not reached, then the next process step takes place. The $h_{1}\left(t\right)$ is treated as an input data point and $h_{2}\left(t\right)$ is created by subtracting the mean of $h_{1}\left(t\right)$ envelope from itself.

(3) $$h_{2}\left(t\right)=h_{1}\left(t\right)-m_{2}\left(t\right)$$

The process is repeated until the function meets IMF criteria. Assuming its being repeated *n* times, $h_{n}\left(t\right)$ is designated $c_{1}\left(t\right)$ and becomes first IMF (labelled further IMF 1). To continue the sifting process, $c_{1}\left(t\right)$ is extracted from the original data x(t), the output of this operation is termed residue $r_{1}\left(t\right)$.

(4) $$r_{1}\left(t\right)=x\left(t\right)-c_{1}\left(t\right)$$

The residue $r_{1}\left(t\right)$ is taken as the original data and the sifting process is repeated, until the final residue $r_{k}\left(t\right)$ is either a constant or a monotonic function. The signal after decomposition is divided into *k* intrinsic mode function and a residue containing information about trend in the data.

(5) $$x\left(t\right)=\sum_{k=1}^{K}c_{k}\left(t\right)+r_{k}\left(t\right)$$

The IMFs have to be extracted one by one, starting from the lowest ones. There is no possibility of extracting a single higher mode without extracting the lower ones beforehand.

#### 2.1.2. Singular Spectrum Analysis

SSA is a nonparametric time series analysis method, which expands on Principal Component Analysis. A comprehensive description of the method and its full mathematical formulation was presented by Golyandina [36]. SSA reduces a signal to a finite number of independent components, ordered according to the variance content in each of them. These components are known as Reconstructed Components (RCs). SSA permits to isolate components of the original signal for better understanding of the phenomena and to obtain characteristic features which may be used for monitoring. SSA is a sequential procedure taking advantage of matrix transformations. In a first step called embedding, a trajectory matrix X is constructed by concatenating a series of lagged vectors. These lagged vectors are derived from the original signal, x(t), according to the parameter *L*—window length. A trajectory matrix X is a Hankel matrix of dimension [L×K], where K=N−L+1, where *N* is the total length of a signal x(t).

(6) $$X=\left[\begin{array}{cccc} x_{1} & x_{2} & \cdots & x_{K}\\ x_{2} & x_{3} & \cdots & x_{K+1}\\ \vdots & \vdots & \ddots & \vdots \\ x_{L} & x_{L+1} & \cdots & x_{N} \end{array}\right]$$

The next step is the decomposition of the trajectory matrix X, which is obtained with the eigenvalue decomposition of the squared matrix $S={\mathrm{XX}}^{T}$ of dimension [L×L]. This decomposition provides a set of eigenvalues in decreasing order $\left(\lambda_{1}\leq \lambda_{2}\leq \cdots \leq \lambda_{L}\right)$ and their corresponding eigenvectors $\left(u_{1},u_{2},\ldots u_{L}\right)$ of S. Thus, the decomposition of S leads to obtaining *L* components, the sum of which results in the original trajectory matrix X. Each individual component $X_{i}$ is defined through an eigentriple (Equation (8)).

(7) $$X=\sum_{i=1}^{L}X_{i}$$

(8) $$X_{i}=\sqrt{\lambda_{i}}u_{i}v_{i}^{T}$$

where

(9) $$v_{i}=\frac{X^{T}u_{i}}{\sqrt{\lambda_{i}}}$$

Principal components are obtained by projecting the trajectory matrix onto the eigenvectors (Equation (10)).

(10) $$p_{i}=X^{T}u_{i}=\sqrt{\lambda_{i}}v_{i}$$

Each individual component matrix $X_{i}$ contains particular information of the original trajectory matrix X and hence, each one contributes more or less towards the reconstruction of X. As the eigenvalues $\lambda_{i}$ are in decreasing order, the first individual component matrices contribute more than the last individual component matrices. In order to reconstruct each individual component, it is necessary to convert the individual component matrices by diagonal averaging. The details of the procedure can be found in [36]. *L* independent RCs are obtained by the decomposition of a signal x(t). Therefore, the original vector signal $x\left(t\right)=(x_{1}\left(t\right),x_{2}\left(t\right),\ldots ,x_{N}\left(t\right))$∈$R^{N}$ is now decomposed into a set of reconstructed components, as shown in Equation (11).

(11) $$x\left(t\right)=\sum_{i=1}^{L}RC_{i}$$

The RCs can be grouped to reconstruct a portion of the trajectory matrix but they can also be used individually to investigate the information included in each one of them. The latter approach is used in this study. It should be noted that each of the RCs can be extracted individually and independently of other RCs.

#### 2.1.3. Parameters of EMD and SSA

Both EMD and SSA are adaptive to the data, but they have intrinsic parameters that need to be defined for a successful decomposition. A fundamental parameter for EMD is the number of sifting iterations (SN) that affects the number of IMFs created, as well as their frequency content [35]. IMFs for experimental pressure signals cover a frequency band located around a central frequency [31]. With a specific number of sifting iterations, EMD works as a dyadiac filter, resulting in the central frequency of each subsequent IMF being half of the previous one [33]. With increasing the number of iterations, the IMF central frequencies are located closer to one another, consequently, the frequency band covered by each IMF becomes narrower and the number of extracted IMFs increases. To retain physical meaning of the modes, one should avoid oversifting. A number of sifting iterations around eight is recommended [35]. A possible benefit of increasing the number of sifting iterations is the narrower frequency range of all IMFs, which may lead to better extraction of the instability feature and decrease the variability of the components. The number of sifting iterations is enforced by the stoppage criterion chosen or can be manually limited to a desired value. In this analysis, the latter approach was used and stoppage criterion was set to ensure that a desired number of sifting iterations can always be performed. The number of sifting iterations was kept constant for each signal portion and set to 8, 16 and 32. Keeping the number of iterations constant did not imply that the same number of IMFs was created for each signal portion in each condition.

The most important parameter of SSA is a window length *L*, affecting the embedding process and the shape of a trajectory matrix. Window length influences the repartition of the data between the components. Increasing the window length, the number of RCs increases, resulting in repartition of the data between more components. To deal with the over-decomposition of the information, grouping of the components is applied [36]. Grouping is discarded in this study, as the pace of detection if of utmost importance and grouping step would definitely increase the overall execution time. In this study, it is validated if a single RC can be used for robust detection and what window length should be used to obtain it. The influence of *L* changes on detection performance are quantified and recommendations concerning the window length and choice of the component are provided.

### 2.2. Strategy for Real-Time Detection of Instabilities

The possible implementation of EMD and SSA into a detection system can be completed in two different ways. One is an online approach, where the new, oncoming data are appended to the previous readouts. It was proposed for both EMD [37] and SSA [38]. The drawback of this method is a possible inertia stemming from existing data, which might negatively impact the pace of detection, important with regards to compressor instability detection. A simpler approach is analysis of subsequent signal portions to understand the performance of the compressor, where each portion is processed separately. The latter method is explored in this study, as it enables clearer understanding of the signal content and allows for more straightforward comparison between the methods.

To understand the sensitivity of the methods, different lengths of signal portions are considered. The signal portion length impacts the time needed for detection in two ways. Firstly, the longer the portion, the longer the acquisition time needed to register it. Secondly, the longer the signal portion, the longer the processing time. The total time of detection is a sum of required acquisition time and processing time. When aiming at a real-time detection, the methods should be capable of processing the data in a time frame similar to or shorter than the required acquisition time as then the compressor operating conditions can be identified before the new portion is collected. The processing time depends on the implementation of the method and hardware used. The comparison in terms of computational time in this study is performed using MATLAB software implementations and a PC computer. It allows benchmarking two methods and provides estimation of the timescale needed for applying each of them, providing some understanding of whether the methods have a potential to be used in real time. However, it might be assumed that if the times of acquisition and processing are similar when using PC, the implementation using FPGA solutions will be at least equally quick [39,40].

As a result of the analysis method applied, a signal portion length *N* is a parameter for sensitivity study for EMD and SSA. Pressure signal obtained from the compressor in most conditions has low signal-to-noise ratio. Therefore, the changes in the signal occurring due to instabilities are not easily detectable. The length of the portion required for a robust distinction of the conditions can vary depending on the method used for decomposition. This study aims at understanding the sensitivity of EMD and SSA and provides comparison of those two methods for the same data set.

### 2.3. Processing of the Components for Detection of Instabilities

There exist several approaches for processing the outcomes of decomposition, for both IMFs or RCs. Research focuses on their energy [31], frequency [21] or amplitude [41], and variation of those values between conditions. It was demonstrated that for detection of instabilities in centrifugal compressors, the approach based on root mean squared values (RMS) of the components can provide insightful results, for both EMD [31] and SSA [29], hence, a similar approach is used in this study. The energy *E* is computed using specific signal portions length *N* expressed in number of data points. Its value is given by Equation (12), where x(n) is a component resulting from original signal decomposition. This is a value representative of a given signal portion of length *N*. The same approach is used for IMFs and RCs.

(12) $$E=\frac{1}{N}\sum_{n=1}^{N}{\left|x\left(n\right)\right|}^{2}$$

A mean energy value for a given component and operating conditions is also used. It is computed according to Equation (13) where *J* is the total number of considered signal portions.

(13) $$\bar{E}=\frac{\sum_{j=1}^{J}E_{j}}{J}$$

**Figure 1:**
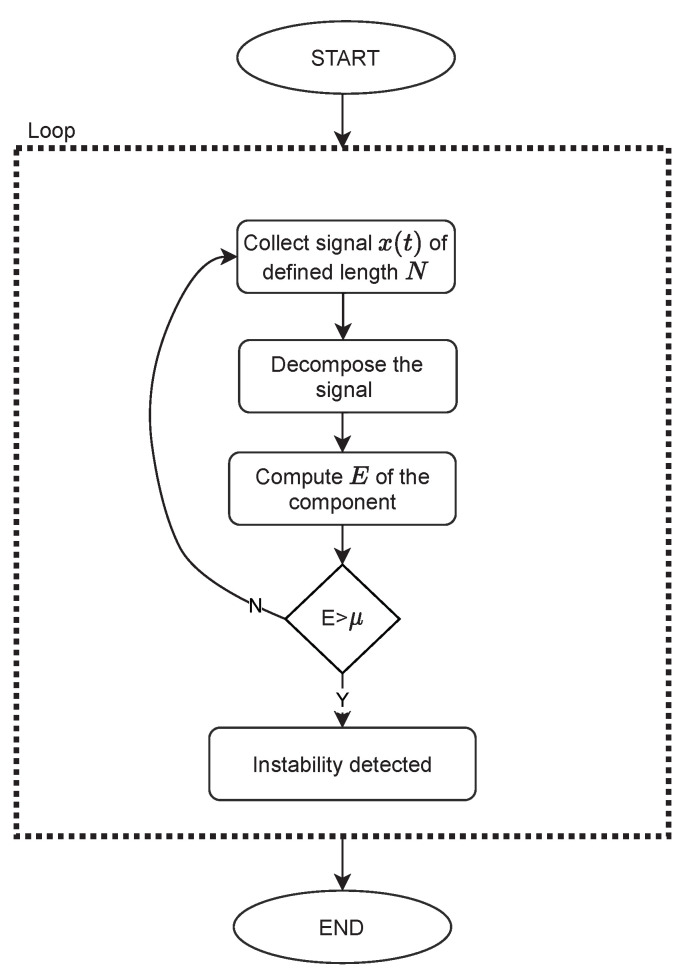
Flowchart of a detection approach.

Detection of instabilities is based on a threshold value μ, obtained as a 99.5th percentile of energy *E* distribution in stable conditions. By formulating the threshold as a high percentile of the data distribution, rather than the maximum value from the set, the outliers can be disregarded. The operating conditions of a compressor are unstable if for a given signal portion the *E* value is above the threshold. The threshold value μ is obtained separately for each set of parameters, including window length, number of siftings and signal length. In practical application, such a system would operate in a loop which would only be interrupted if a detection takes place. The flowchart of the approach is presented in Figure 1.

### 2.4. Test Rig and Pressure Signals

**Figure 2:**
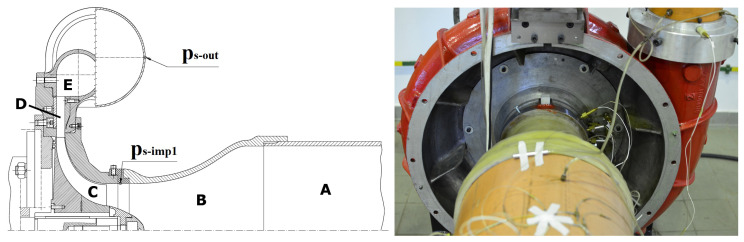
Centrifugal compressor under investigation; cross-section of the machine and photo of the test rig.

Pressure data were obtained from two locations inside the centrifugal compressor shown in Figure 2. The air was supplied to the compressor through an inlet pipe (A) and Witoszynski nozzle (B), which accelerated the flow before the impeller (C). Downstream of the rotor, air passed through a vaneless diffuser (D), and a circular volute (E). The flow was afterwards directed into an outlet pipe, at the end of which throttling value was mounted.

The sampling rate of 100 kHz was used, allowing to capture a wide range of flow structures. The test stand was equipped with dynamic subminiature Kulite transducers connected to an Iotech Wavebook 516/E data acquisition system. The natural frequency of the sensor was 1500 kHz, significantly above the data acquisition frequency. The data from this machine were previously analysed and described [1,9], therefore, it can efficiently be used for benchmarking of different methods.

For described investigation, data for different operating conditions were considered. The conditions were imposed through throttling at the outlet of the machine. The throttling level was defined through throttle opening area (TOA), being the relation of the open channel area to its total area. To obtain data for given operating conditions, the throttling level was set to the desired value of TOA, and pressure recording of over 2 million data points (over 20 s) was made. It was repeated for each TOA of interest to map the whole operating range. Consequently, over 2 million data points were available for each TOA value. For this study, TOA values used ranged from 35%, where the machine was working in a stable regime to 8.5%, where deep surge was present. In between those values, a transition region, as well as a local instability—inlet recirculation were observed. Table 1 summarizes TOA values subjected to analysis in this study and observed flow conditions.

The presence of inlet recirculation was observed for the sensor $p_{s-imp1}$ located upstream of the impeller. Surge had the strongest signature for the $p_{s-out}$ sensor at the outlet [9]. Therefore, sensors from these two locations were used in the study—$p_{s-imp1}$ for inlet recirculation and $p_{s-out}$ for surge. For each TOA value, 20 s of the signal were collected, resulting in over 2 million data points.

The decomposition with EMD and SSA is performed on non-overlapping signal portions of length *N* extracted from a longer signal. Overlapping was not considered in this study as authors aimed at assessing the original method’s performance and its change with intrinsic method parameters. Using the overlap could also negatively influence the pace of detection as the same portion of data would have to be decomposed more than once. Varying length of the portions is used to evaluate the sensitivity of the methods. For each length, the accuracy of prediction is demonstrated to understand its effect for both methods. This allows to establish how sensitive a method is to the appearing instability and how quick a detection could be made. The sensitivity is validated for signal portions *N* = 1000, 5000, 10,000 and 50,000 samples, equivalent to 0.01, 0.05, 0.1 and 0.5 s of wall clock time or 1, 5, 10 and 50 revolutions of the impellerm respectively. A total of 40 windows were used for each operating point to account for the stochastic character of the pressure signal.

## 3. Results

The results are divided into two sections according to the instability that is to be detected. The first one discusses the sensitivity of the methods for inlet recirculation and the second one demonstrates how well surge can be detected with EMD and SSA.

### 3.1. Overview of the Signals

**Figure 3:**
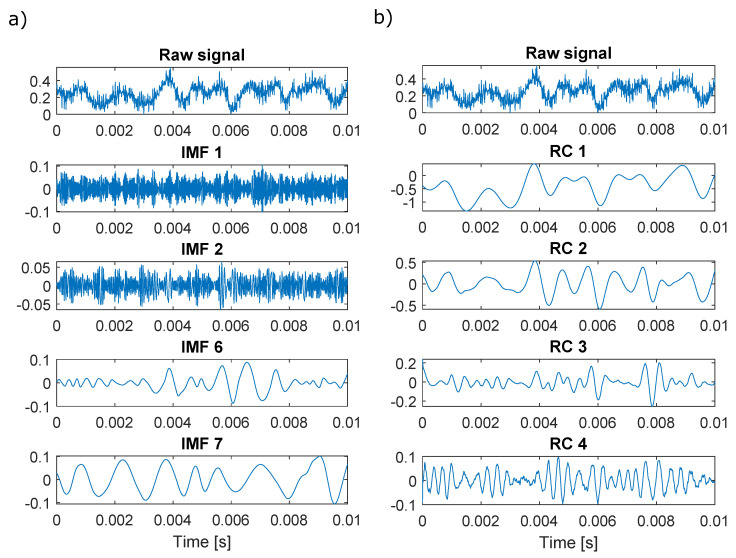
Raw signal and selected components from its decomposition with (**a**) EMD and (**b**) SSA at TOA 25% for $p_{s-imp1}$ outlet sensor.

To understand the character of the analysed data and the decomposition, a raw signal along with selected IMFs resulting from decomposition is presented in Figure 3. It can be noticed that first IMFs and first RCs differ significantly in terms of signal frequency. For EMD, each subsequent IMF is of lower frequency, while for SSA, the RCs do not have to be ordered by the frequency. For EMD, the IMFs should be zero-mean simple harmonic oscillations [33], while in SSA, the components can exhibit any behaviour. IMFs 6 and 7 have similar frequencies and amplitudes in the selected signal, which may indicate the possibility of mode mixing. RCs 3 and 4 show beating-like behaviour, which suggests that the signal character changes importantly for the same operating conditions and within a short time contained in the presented time frame.

### 3.2. Inlet Recirculation

Inlet recirculation is often the first instability appearing when the mass flow of the compressor decreases, preceding the onset of surge [6]. It forms near the leading edge of the impeller blade, at the inlet to the compressor. It has a form of a recirculating zone of fluid, which might be local or occur around a whole annulus of the compressor and change its size with varying mass flow [42]. Inlet recirculation is responsible for increased loss in the compression system [6] and can be regarded as an early symptom of approaching surge [29]. Thus, its detection is important from the perspective of economy and can be taken advantage of for early detection of surge onset. Inlet recirculation in the studied compressor was manifested in a pressure signal by a broadband noise of frequency around 1000 Hz, but with no dominating frequency discovered [9]. It had the strongest signature in the signal from the sensor located on the shroud before impeller. Therefore, the data from this sensor were used for the study of EMD and SSA.

#### 3.2.1. EMD-Based Detection

**Figure 4:**
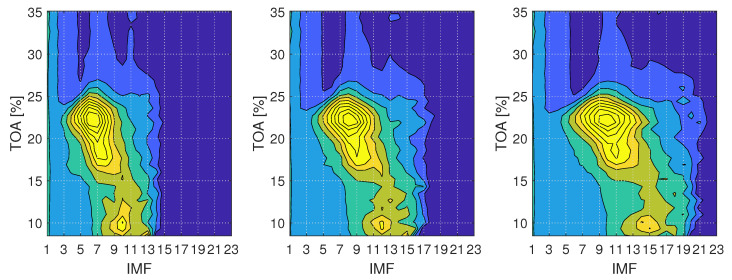
Mean energy $\bar{E}$ of dynamic pressure signal from $p_{s-imp1}$ sensor before impeller for signal portions of length *N* = 10,000; (**left**) 8 sifting iterations, (**middle**) 16 sifting iterations, (**right**) 32 sifting iterations.

EMD decomposes the signal into a number of IMFs, the content of which depends on the input signal and decomposition parameters. The choice of IMFs which can be used as indicators is not straightforward and requires prior knowledge of the system dynamics or thorough analysis. In this study, the choice of IMFs was made based on analysis of mean energy changes of IMFs. Those changes are presented in Figure 4, where the mean energy $\bar{E}$ of IMFs is plotted for different number of sifting iterations and operating conditions. The values are obtained for each IMF and TOA value and interpolated in between to provide a comprehensible visualisation of the changes. With the energy of IMFs being linked to the presence and intensity of instabilities [31], increased energy regions allow to understand which IMFs hold features of instabilities.

Inlet recirculation can be observed as an increase in energy value for a number of IMFs, with peak location changing for varying number of sifting iterations. When the number of sifting iterations is set to 8, the strongest trace of inlet recirculation is held by IMF 6. Increasing the number of siftings to 16 and 32, the peak shifts to IMF 8 and IMF 10, respectively. The overall number of meaningful (non-zero) components also increases.

**Figure 5:**
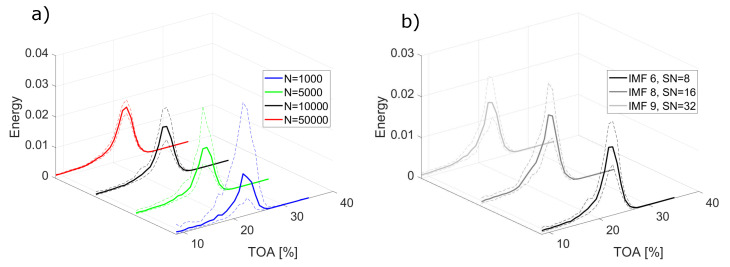
Mean energy $\bar{E}$ (solid lines) and confidence interval (dashed lines) for different method parameters: (**a**) varying signal length *N* for SN = 8; (**b**) varying number of siftings for *N* = 10,000.

**Figure 6:**
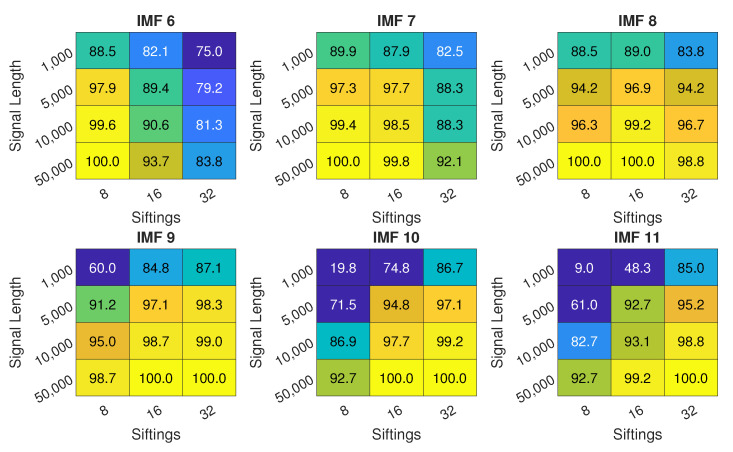
Inlet recirculation detection accuracy for IMFs 6 to 11 with different signal length *N* and different sifting iterations number; colorscale is used only for values above 75% for easier interpretation.

Figure 5 depicts the influence of signal length and number of sifting iterations on the mean value and dispersion of selected IMFs. The selection was based on the best performance for inlet recirculation detection (Figure 6). Performance is understood as the number of correct detection of unstable conditions to the total number of signal portions considered for those conditions. The length of the signal in the analysed range does not impact much the mean energy value across TOAs, as shown in the example of IMF 7 obtained for different signal lengths in Figure 5a). Even for the smallest *N*, the mean value captures well the presence of inlet recirculation. What differs is the dispersion of the data, which is the highest for the shortest signal length and in general decreases with increasing *N*. The dispersion also varies with TOA, being relatively small in stable conditions and increasing as the inlet recirculation sets in.

Figure 5b demonstrates the mean and dispersion of the data for best-performing IMFs taken for different number of sifting iterations. The overall shape of the distribution is similar in a whole operating range. The number of sifting iterations does not seem to have influence on the dispersion—neither positive, nor negative. For the chosen approach to detection, the changes in number of sifting iterations in the analysed range do not affect the mean value or dispersion of the data, thus, they should have no influence on the sensitivity of the method.

The performance of EMD for varying signal length *N* and number of siftings SN is presented in Figure 6. The performance is the lowest for the shortest signal, but still can reach close to 90% for selected IMFs and number of siftings. Increasing signal length results in improvement of the performance. Over 95% accuracy can be reached for *N* = 5000, over 99% accuracy for *N* = 10,000 and 100% for *N* = 50,000. An important observation is that the best-performing IMF changes with the number of siftings. For SN = 8, IMF 6 provides the best performance, with IMF 7 being only slightly worse. For SN = 16, the best is IMF 8, with 7 and 9 following closely. For SN = 32, IMF 9 is the best, closely followed by 10 and 11. The overall trend is that the feature of interest is pushed towards higher IMFs due to IMFs having narrower frequency spectrum.

#### 3.2.2. SSA-Based Detection

The inlet recirculation can also be captured by using changes in energy of RCs produced by SSA, as was shown by Logan et al. [29]. In that study, RC 2 was used for extraction of inlet recirculation features in centrifugal compressors. The optimal window length proposed for using RC 2 was 80, but the optimum was defined using difference in RMS between the peak of inlet recirculation and the reference conditions. However, the most prominent peak at the maximum does not guarantee the best identification across the whole region of instability, especially considering the dispersion of the data.

**Figure 7:**
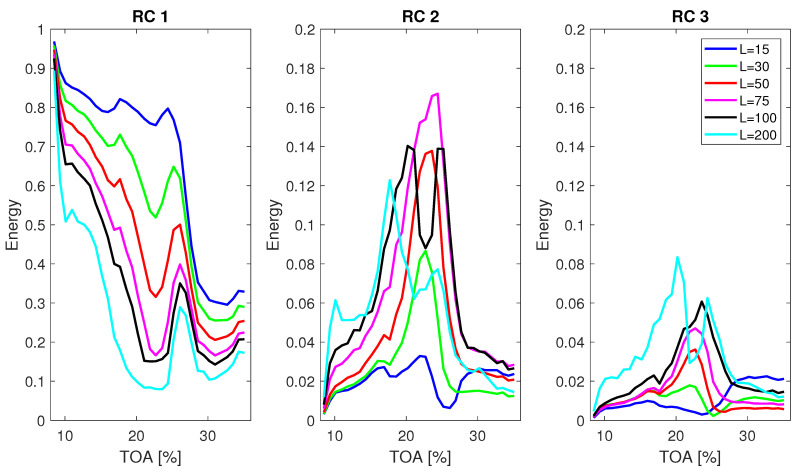
Mean energy $\bar{E}$ of RCs from 1 to 3 over different operating conditions for varying window length and signal length *N* = 10,000.

The influence of window length *L* on the mean energy $\bar{E}$ for the first three RCs is presented in Figure 7. Only first RCs are shown, as it is expected that instabilities will be expressed in the components with high variance. The window length *L* varied from 15 to 200 data points and computations were performed for signal length *N* = 10,000. Depending on the window length, the traces of inlet recirculation are clearly present in RCs 2 and 3. Some effects of recirculation appear even in RC 1 for the almost all values of *L*. RC 2 isolates well the inlet recirculation for *L* = 30, 50 and 75. For higher values of *L*, a more consistent indication of recirculation can be obtained from higher components, as the behaviour of energy is more consistent throughout the TOA range. The drop in $\bar{E}$ of RC 2 for *L* = 100 is not expected, as the recirculation intensity was high in that region [9]. This implies that some of the energy from the phenomenon was passed to a different RC.

By choosing the window length, changes are introduced to the decomposition. These changes are more complex than those caused by changing the number of sifting iterations for EMD. There are as many components created as the window length *L*, therefore increasing *L* leads to decomposing the signal into more components. Consequently, the feature contained in a specific RC in case of lower *L* value can be shared between two or more RCs for a higher *L* or shifted to different RCs. RC 2 energy chosen as a feature representing inlet recirculation is only useful for specific window lengths. When the window is too short, the separation of signal into components is not sufficient and features of interest are poorly separated. For the shortest window, most of the energy is captured by RC 1. The energy of RC 1 for the inlet recirculation region decreases with increasing window length, as the features are better separated. When the *L* is over 30, the inlet recirculation peak gets shared between the RC 2 and RC 3 which is not desired if a single RC is to be used for monitoring.

**Figure 8:**
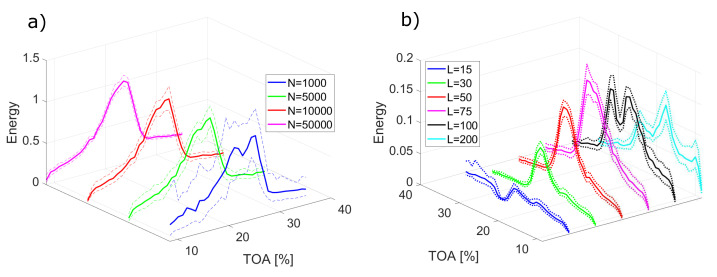
Mean energy $\bar{E}$ (solid lines) and confidence interval (dashed lines) for different method parameters: (**a**) varying signal length *N* for *L* = 50; (**b**) varying window length *L* for *N* = 10,000.

For changing signal length *N* with constant window length *L* (Figure 8a), the mean value of energy $\bar{E}$ remains similar, while dispersion grows with increasing signal length, similarly as for EMD. The changes in window length for the same signal length (Figure 8b) affect both mean value and dispersion. No clear relation of dispersion to varying window length is present. Contrary to EMD-based components, the absolute value of dispersion remains similar throughout the analysed range of TOA.

**Figure 9:**
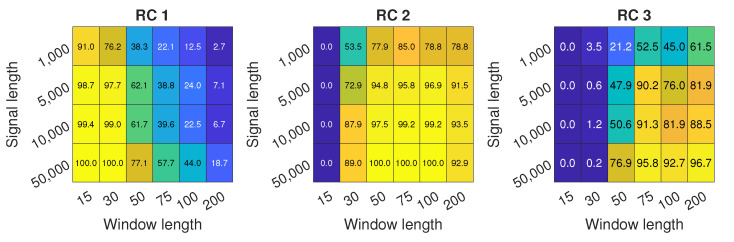
Inlet recirculation detection for RCs 1 to 3 with different signal length *N* and different window length *L*.

Figure 9 presents the detection potential for all of the combination of parameters. It can be shown that RC 2 performs well in a range of windows from 50 to 100. RC 1 can be used as a measure of inlet recirculation for the shortest window lengths, but it provides no differentiation between inlet recirculation and surge, which can be deducted from Figure 7. For the longest windows, the response of RC 3 to appearance of IR is also visible, but it is comparable to RC 2 only for *L* = 75. For most parameters, shortest signal length does not provide a satisfactory level of detection, reaching close to 80% for most of the window lengths. For *N* = 5000, over 95% accuracy can be reached, and for *N* = 10,000, almost 100%. Similarly as for EMD, the longer the signal, the higher the accuracy of detection.

### 3.3. Surge

Surge detection is of utmost importance for the safety of every compressor. Term surge refers to low-frequency pressure oscillation of the fluid that may lead to high-amplitude vibrations of the machine, resulting in its destruction [3]. Surge onset may happen abruptly, therefore, fast surge detection is a must for safety reasons. In this study, the potential of quick surge detection is evaluated by exploring the possibility of quick and robust differentiation of stable and unstable conditions, including both mild and deep surge, as per Table 1.

#### 3.3.1. EMD-Based Detection

**Figure 10:**
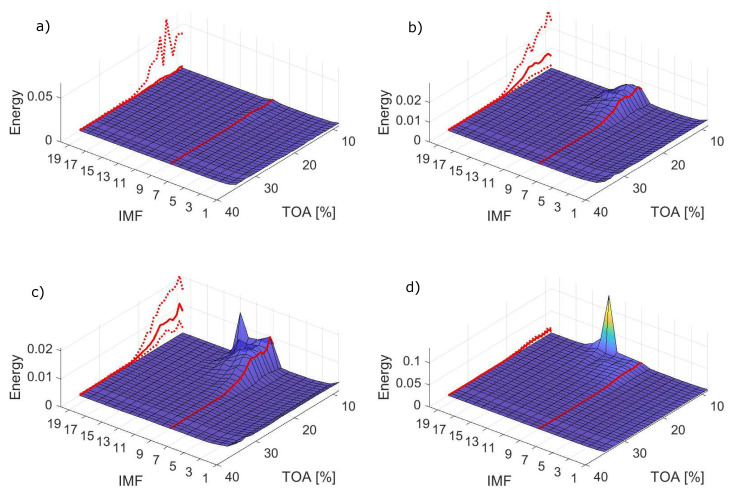
Surface plot of energy in the studied range for varying signal lengths: (**a**) *N* = 1000, (**b**) *N* = 5000, (**c**) *N* = 10,000, (**d**) *N* = 50,000; solid line represents the mean energy of IMF 9, marked with red line on the surface plot and dashed lines represent energy confidence intervals.

An important and non-trivial issue is the choice for IMF to be used for constructing a detection feature. IMFs are ordered by decreasing central frequency, therefore, for surge manifesting at low frequencies and high sampling frequency for the analysed system, it is expected that higher IMFs would hold the desired information. Surface plots of energy for all signal lengths obtained for number of siftings SN = 8 are shown in Figure 10.

**Figure 11:**
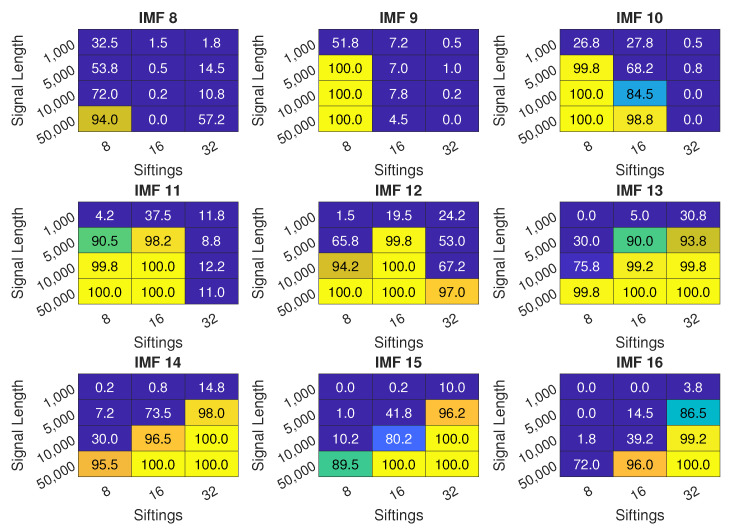
Surge detection accuracy for IMFs 8 to 16 with different signal length *N* and different number of siftings.

IMF 9 with the highest detection potential, defined with results from Figure 11, is highlighted in red and projected onto the side of the plot along with its dispersion. For the shortest signal (*N* = 1000) surge is not captured well. An increase in RMS of IMF 9 is observed, but it is not consistent for the whole unstable region. A more consistent indication is yielded from all the longer signals. With *N* = 5000, the important increase in IMF 9 is noted along with higher IMFs. For *N* = 10,000 the differences between stable and unstable conditions are more significant and some traces of deep surge are getting captured at the end of the TOA range. For the longest signal *N* = 50,000, a deep surge peak visible for IMF 13 begins to dominate the energy distribution. The dispersion of the energy for IMF 9 is similar to what was observed for EMD with inlet recirculation—the longer the signal portion *N*, the lower the dispersion. The dispersion also grows with increasing levels of instability. For the shortest signal, in the highly unstable range, the lower values of dispersion are almost zero or equal to zero. This stems from the fact that for some signal portions, the decomposition was stopped before IMF 9 was obtained. The effect of varying the number of sifting iterations is not explicitly presented, but it is similar to what was observed in case of inlet recirculation, which can be deduced based on the results presented in Figure 11.

Depending on the number of siftings, different IMF work best for detecting surge. For 8 siftings—IMF 9, for 16 siftings—IMF 12, while for 32 siftings—IMF 14. The shortest signal length does not provide good detection performance for surge, barely exceeding 50%. However, from *N* = 5000 inclusive, a 100% accuracy can be obtained for the IMF 9 with SN = 8. For each value of SN, an IMF with over 99% accuracy can be defined. Overall, very good detection performance is offered by EMD, provided that a correct IMF is chosen.

#### 3.3.2. SSA-Based Detection

**Figure 12:**
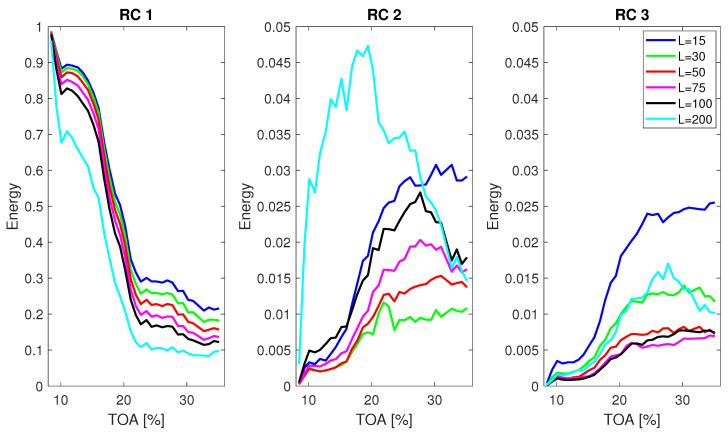
Mean energy $\bar{E}$ of RCs from 1 to 3 for varying window length and signal length *N* = 10,000.

For surge detection with SSA, similarly as for inlet recirculation, the appropriate component to be used has to be defined. Figure 12 presents the influence of window length for RC 1 to RC 3 for a signal portion *N* = 10,000. For RC 1, the behaviour for all window lengths is similar, reacting to appearance of unstable structures and surge. The increase in value is present from the very limit of the TOA range (35%), but the slope changes importantly at around 20%. Therefore, regardless of the window length, the basic differentiation between stable and unstable is possible. A downward shift in absolute value is visible with increasing window length, coming from the energy being spread in between the higher number of components.

For RC 2, the relation for changing window length is not that structured. For the two shortest window lengths, their value almost monotonically decreases with decreasing TOA—the majority of energy is transferred to RC 1 as the instabilities appear in the flow. For *L* from 50 to 100, the energy level is stable for the most part and decreases for the region of surge instability. For the longest window *L* = 200, the increase in energy of RC 2 is present for the mild surge region and a drop takes place when deep surge occurs. Only for this window length, the separation of components is sufficient to distinguish mild from deep surge. The overall shape of the mean energy is not as smooth as in case of EMD. It might be attributed to variations in the frequency content of RCs and transfer of energy in between the components.

**Figure 13:**
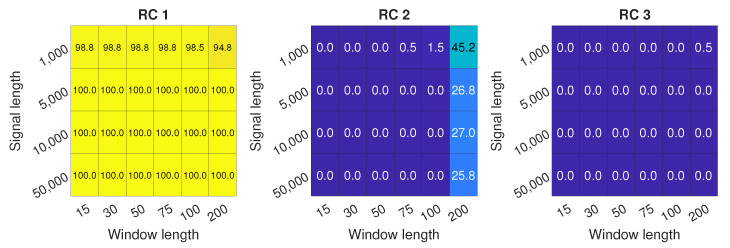
Surge detection accuracy for RCs 1 to 3 with different signal length *N* and different window length *L*.

Figure 13 summarizes surge detection potential of selected RCs. The detection with RC 1 is the best, as almost for every set of components, 100% detection rate is obtained, even for the shortest signal length. RC 2 demonstrates some potential with the longest window, which stems from the increase in the mild surge region. However, the overall detection rate for RCs is below 50%. RC 3 shows no detection potential with the approach from this study.

### 3.4. Timing of the Methods

**Figure 14:**
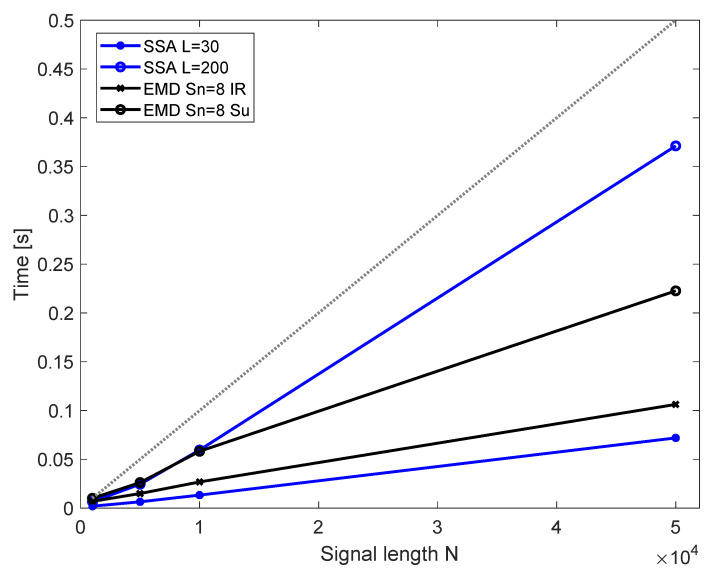
Time of obtaining the components with EMD and SSA for selected parameters for EMD and SSA; dotted line represents the time needed for acquisition of *N* data points.

The processing time is crucial in compressors instabilities detection. Figure 14 summarizes the times of processing for signals of different length and different method parameters in comparison to the acquisition time. Presented results were obtained with use of a PC class computer (6 core processor at 3.6 GHz) and MATLAB software. The decomposition was ran 40 times for each of the signal lengths *N* to obtain a mean value, presented in the plot. The timing data are demonstrated to provide an estimate of a processing time needed for each method and compare them using the same computational setup.

For EMD, the number of sifting is kept at 8 as no benefit of increasing this number was observed. Detection of inlet recirculation requires computation of 6 IMFs and detection of surge requires obtaining 9 IMFs. The time for both of those remains below the acquisition time line. For SSA, it is assumed that only a single, selected RC is obtained. Then, the window length is important, as it influences the computational effort for decomposition. In case of SSA, timing for the selected window (*L* = 75) and longest investigated window (*L* = 200) is below the data acquisition time. The time needed for processing increases almost linearly with signal length. The longer the signal, the larger the time margin between processing time and acquisition time.

The timing is highly dependent on the machine but also on software implementation. It was demonstrated that quick processing of the signal can be achieved with use of FPGA architecture [39,43] and this approach would be favourable when constructing the solution managing a physical machine. It is expected that the processing times with a specially designed architecture and optimized software implementation would be similar to or lower than those of a PC.

## 4. Discussion

Detection of different aerodynamic instabilities is possible with EMD and SSA, which was shown previously in literature [29,31]. This work proves that both of those methods have high sensitivity and can provide indication of operating conditions using short signal portions. A very accurate detection for most of the instabilities and methods can be achieved using a signal portion corresponding to 0.1 s. The processing time of such a portion is shorter than required acquisition time, making it possible for the methods to operate in real time. Therefore, both EMD and SSA might be considered prospective candidates for a quick or even real-time instabilities detection system.

To construct a successful detection system, intrinsic decomposition parameters have to be set and proper decomposition components have to be chosen. The influence of the intrinsic methods parameters, being number of sifting iterations for EMD and window length for SSA, was investigated for the compressor pressure signal in the context of instabilities detection. In case of EMD, the increase in number of siftings did not provide any advantage over the base value. The IMF of interest had a higher sequential number as all IMFs held narrower frequency bands. Increasing the number of sifting iterations increases the time needed for decomposition and the number of IMFs that have to be extracted before the IMF of interest is obtained. Therefore, it is recommended to keep the number of sifting iterations low, as suggested by Wang [35].

The influence of window length for SSA has more dimensions to it, as it affects the repartition of energy between RCs in a non-trivial manner. With increasing window length, the number of components increases and features of the signal become more separated. A long window can lead to over-separation of a feature related to instability into more components, which results in the need of grouping. Grouping could provide more accurate results, but requires even more thorough analysis of the system and adds a number of adjustable parameters, making such an approach less general. The choice of window length remains case-specific, however, the recommendations made in reference to the compressor geometry, rotational speed and sampling frequency [29] should be further explored to observe how universal they prove to be for other machines.

For both methods and both instabilities, the mean value of energy was a very good indicator of conditions. When examining the robustness of detection based on separate signal portions, the dispersion of the energy was considerably affecting the results. For EMD, the dispersion was high for the unstable region, but remained low in stable conditions. In the case of SSA, it was similar throughout all conditions, being smaller than that of EMD in unstable regions, but larger in stable regions. For both methods, the longer signal portion length *N* resulted in lower dispersion. With the instabilities’ intensity changing over time for the same TOA value [44], data dispersion is unavoidable. Higher dispersion for EMD in the unstable region can be caused by mode mixing, a phenomenon inherent to EMD [34]. The transfer of energy from one IMF to another leads to high variation of energy for a chosen IMF. One should be aware that for some specific cases, it might be possible that the dispersion of the data will be unfavourably increased by the decomposition method. Mode mixing could be diminished by using improvements to EMD, such as Ensemble Empirical Mode Decomposition [22,45] or Partly Ensemble Empirical Mode Decomposition [46]. Both of those methods can deal with mode mixing, however, their application, based on EMD, requires several decompositions, therefore, it is not suited for quick detection. Despite possible mode mixing, EMD displayed high sensitivity and accuracy of instabilities detection for the test compressor, and should be capable of similar performance for other machines.

EMD and SSA differ in principles of operation, as EMD is based on the waveform of the signal, while the SSA takes advantage of covariance of the trajectory matrix build from the input. For a compressor signal, it translates into more predictable content of the EMD components and less predictable content of SSA components. In case of EMD, the subsequent components represent adjacent frequency bands. For a multi-component signal as that from the compressor, the central frequency of a specific component remains relatively constant throughout the operating conditions. Therefore, if the early symptoms of inlet recirculation are present in the IMF 6, this IMF will hold recirculation features for all compressor operating conditions. SSA orders the components according to their variance in respect to the signal [36]. Therefore, the first component will hold the highest variance part of the signal, which in case of unstable operation will represent the dominating phenomenon or a piece of it. RC 2 will contain the components of second-largest variance, RC 3 third, and so on. What is represented by a selected RC depends strongly on the structure of the signal, which changes importantly throughout the compressor operating range. There is no guarantee that for the same machine, a selected RC will always represent the same phenomenon, especially if the dominating phenomenon changes over the range of operating conditions.

In case of the sensor $p_{s-imp1}$ used for inlet recirculation detection, it can be seen that RC 1 holds the signature of surge, as it increases for the lowest TOAs. However, it also seems to capture some traces of inlet recirculation, as before the TOA where the peak of recirculation occurs, the increase in energy is observed. It might be possible that for a different machine with stronger inlet recirculation signature, its effects would affect RC 1 more and would not be so clearly present in RC 2. With EMD, inlet recirculation features are shared between several components, however, after defining the one to trace it remains connected to that component. Knowing a possible frequency range expected for recirculation, one can define which IMF to trace using stable conditions only. From a physical point of view, there is less risk of unexpected changes in component energy and one can be certain that it only reacts to the appearance of inlet recirculation. Therefore, EMD providing higher stability and confidence of detection can be considered a better choice for inlet recirculation.

For surge, the same principles of each method provide different advantages and disadvantages. With EMD, one can observe that signature of mild surge is held by different IMF than deep surge. What is more, these are high IMFs that require previous ones to be extracted. Due to the large number of IMFs extracted before and changing structure of the signal, the high IMFs are more prone to appearance of mode mixing, which increases the dispersion of the data. The choice of a proper IMF is also not trivial and the accuracy of detection is dependent on sifting parameters, as well as sampling rate, as it would directly influence the waveform of the signal. For SSA, surge, either mild or deep, is expected to be a dominating mode in the pressure signal. Having the most important variance, it is to be captured in the RC 1. Therefore, by choosing the RC 1 for detecting surge, the overall stability of the system can be monitored. The influence of the intrinsic parameters and signal length (Figure 13) is also much lower than in case of EMD. Therefore, due to the much easier choice of components and quicker detection possibility, SSA is considered a better method for efficient surge detection.

## 5. Conclusions

In this paper, the operation of non-linear signal processing methods—EMD and SSA—were investigated and compared for the purpose of quick aerodynamic instabilities detection in centrifugal compressor. Based on energy of selected components, the overall detection possibilities and signal length needed for robust identification of inlet recirculation and surge were compared. It was shown that:

- Both EMD and SSA offer high sensitivity of detection, requiring 0.1 s of data acquisition time for over 99% accuracy in differentiation of the conditions. The data processing time using PC class computer was around 0.05 s. With processing time lower than required acquisition time, both methods have potential to be used in real-time detection systems.;
- When only a single component is of interest, SSA operates quicker than EMD. This pace advantage comes from that SSA allows to independently obtain each of the components, unlike EMD, which is sequential and requires all preceding IMFs to be extracted. With both methods, there should still remain space to increase the number of extracted components, while remaining below the acquisition time limit;
- EMD can be considered a better method for inlet recirculation detection, as its components are more interpretable and confined to physical phenomena than those of SSA, especially if the phenomena are not dominating in the signal. Thus, a selected component can be associated with inlet recirculation with higher confidence. SSA can be regarded better for surge detection as it is faster than EMD and the most important instability is expected to be found in RC 1;
- The number of sifting iterations has little influence on the overall performance of EMD. Keeping the value of iterations low ensures quick decomposition and does not decrease the accuracy of detection compared to a larger number of siftings. Choice of window length for SSA has more important influence on the outcomes of decomposition, especially if the feature of interest is not the only one dominating in the signal. If a dominating feature is to be extracted, the window length has less influence;
- Both methods rely on low frequency to make a detection, disregarding the high-frequency components. Therefore, it should be possible to obtain similar indications with lower sampling frequency. With the same acquisition time, the processing time could be shorter, making both SSA and EMD even more adequate for quick and robust detection of instabilities.

## Figures and Tables

**Table 1:** TOA values and corresponding operating conditions.

TOA [%]	General Condition	Detailed Condition
5–8.5%	Unstable	Deep surge
12–17%	Unstable	Mild surge
18–26%	Transient	Inlet recirculation
27–35%	Stable	Optimum performance

## Data Availability

Data available on request from authors; please contact Grzegorz Liskiewicz grzegorz.liskiewicz@p.lodz.pl.

## Abbreviations

The following symbols and abbreviations are used in this manuscript:

EMD	Empirical mode decomposition
SSA	Singular spectrum analysis
RC	Reconstructed component
IMF	Intrinsic mode function
TOA	Throttle opening area
RMS	Root mean square
PC	Personal computer
FPGA	Field-programmable gate array
